# Associations between adolescents watching pornography and poor mental health in three Swedish surveys

**DOI:** 10.1007/s00787-022-01992-x

**Published:** 2022-05-07

**Authors:** C. G. Svedin, M. Donevan, M. Bladh, G. Priebe, C. Fredlund, L. S. Jonsson

**Affiliations:** 1 Department of Social Sciences, Marie Cederschiöld University, Stockholm, Sweden; 2 Department of Biomedical and Clinical Sciences, Linköping University, Linköping, Sweden; 3 Department of Psychology, Lund University, Lund, Sweden; 4 Department of Social and Psychological Studies, Karlstad University, Karlstad, Sweden

**Keywords:** Adolescents, Watching, Pornography, Mental health, SCL-25, TSCC

## Abstract

The aim of this study was to examine the association between watching pornography and poor mental health in three repeated cross-sectional surveys in Sweden (2004, 2009, 2014) among high school seniors (13,277 students) with an average age of 18 years. The same index questions concerning ever having watched pornography and the frequency of watching pornography during the last year were combined with three different measures of psychological health and background control variables in multiple logistic regression and forward stepwise logistic regression models. The repeated cross-sectional surveys did not find any consistent associations across years between poor mental health and ever having watched pornography or the frequency of watching pornography. Having watched deviant pornography (containing violence, children and/or animals) was associated with poor mental health among boys in two surveys but only in one survey among girls. Other characteristics, such as mother’s unemployment (especially boys), parenting style (especially high controlling parents among boys) and experiences of sexual abuse (especially penetrating abuse among girls), were more consistently and strongly associated to poor mental health across the three surveys. This study stresses the importance of controlling for multiple background variables when studying the association between watching pornography and mental health, since the association might primarily be explained by underlying confounding variables.

**Supplementary Information:**

The online version contains supplementary material available at 10.1007/s00787-022-01992-x.

## Introduction

Globally, a growing body of literatures have focused on the lifetime incidence and the frequency of pornography use among young people. In a review of surveys published between 1995 and 2015, researchers Peter and Valkenburg [1] conclude that while it is common for young people to watch pornography, figures vary greatly between studies. Young people can be unintentionally exposed to pornographic materials or intentionally seek out pornography [1]. Unintentional exposure refers to instances when young people inadvertently stumble upon pornography, where exposure to pornography is often undesirable. This could for instance occur when opening an unsolicited email, entering an incorrect web address, using an internet search engine, or being exposed to pop-up images containing pornographic materials and sex advertisements. The prevalence of unintentional exposure ranges anywhere from 19% in a US study among children aged 10–12 [2], to 25% of 11–16-year-olds in the UK [3], and up to 60% and 84% of 16–17-year-old Australian boys and girls respectively [4]. Some argue that encountering pornography is inevitable, given the pervasiveness of sexualized materials in media [5]. When it comes to young people's intentional use of pornography, this has typically been defined as voluntary and/or purposeful, and often involves active exploration. The prevalence also varies considerably from study to study. For instance, Ybarra and Mitchell [6] found that only 7% of 10- to 17-year-olds in USA were intentional users in contrast to Chen et al. [7] who reported as many as 59% of Taiwanese students in grades 10–12 had intentionally used pornography within the past year. Studies among adolescents in Sweden have in general found that almost all young boys and around half of all girls watch pornography at least once or twice a year [8]. Generally, the differences found across studies in pornography use can be explained by the fact that watching pornography increases with age and varies by sex [3], and that internet pornography offers users accessibility, affordability, and anonymity [9] and children and young peoples’ increasing ownership to smartphones and iPads [10].

With much of the literature focusing on young people’s exposure to and use of pornography, there have been increasing concerns about the consequences that pornography might have on young people's psychosocial health. There are a number of ways that pornography could affect a young person’s psychological wellbeing. The predominance of body enhancements may negatively affect adolescent’s perceptions about their bodies, appearance, and/or sexual performance. Further, the pervasiveness of harmful gender stereotypes and men’s violence against women [11] could lead to relationship dysfunction and increase the risk of intimate partner violence, and thus impact mental health. Finally, researchers have observed how the rise in new technologies create favorable conditions for addictive behaviors such as compulsive pornography use [12], which could contribute to lower life satisfaction and self-esteem, and lead to higher social isolation. That said, the direction of the relationship between poor mental health and pornography watching is unclear. Theoretically, poor mental health could affect a person’s pornography use where pornography is used as a way to "cope" with depression and mental health problems [6]. The frequency of pornography use, the age of consumption, and the sex of the viewer are all variables that could influence the relationship between pornography psychosocial health [13].

Most studies focusing on young people and pornography have looked at the relationship between pornography consumption and sexual attitudes and behaviors, in particular sexual risky behavior [14] and sexual aggression [1]. Fewer studies have specifically focused on the association between young people’s pornography consumption and psychosocial health. Among existing studies, researchers have found associations between pornography consumption and less social interaction and bonding [15], lower self-esteem [16, 17] and more often symptoms of depression [18, 19]. Mattebo et al. [20] found that boys who frequently (daily) watched pornography (10%) spent significantly more time at the computer (more than 10 h straight several times a week) and reported more problems with relationships with peers, truancy, obesity and use of both alcohol and tobacco. On the other hand, their self-reported health, apart from being overweight, was no worse than those who did not watch pornography frequently. In a later study, pornography use among girls was associated with similar outcomes, such as more sexual experiences, peer problems, alcohol, and tobacco use [21]. A longitudinal school-based study conducted by the same research group showed that psychosomatic symptoms at the age of 18 were predicted by high pornography consumption at the age of 16 in combination with being a girl, living with separated parents and attending a vocational high school program [22]. On the contrary, depressive symptoms at age 18 were predicted by less pornography use at age 16 and being a girl. A cross-sectional study in six European countries concluded that exposure to pornography is ubiquitous, more relevant to boys, and is associated with both positive/competences and externalizing behavior problems [23]. In Svedin and colleagues' survey from 2004 [24] boys who consumed pornography "more or less daily” consumed alcohol more often than other male adolescents—a behavior shown to be associated with psychological distress, such as anxiety and depressive symptoms [25]. In the most recent publications on the subject, a six-wave latent growth and latent class modeling approach failed to corroborate the notion that pornography use in middle to late adolescence contributes to adverse psychological well-being (self-esteem and depression/anxiety) [26, 27]. However, the authors were unable to rule out a relationship between pornography and adverse psychological well-being among adolescents at an earlier developmental phase, particularly among female adolescents.

Even if Alexandraki et al. [28] found that a majority of their reviewed studies reported symptoms of depression and behavior problems, results on the relationship between young people’s pornography use and poor mental health are inconsistent and thus inconclusive. To further contribute to our understanding of this relationship, this study aimed to examine the association between watching pornography and poor mental health in three repeated cross-sectional surveys in Sweden (2004, 2009, 2014) [29–31] among high school seniors, in total 13,277 pupils with an average age of 18. What is novel in this study is the approach that in three different studies with identical questions about pornography use but with different questionnaire for mental health, gives the possibility to study whether there is a consistent association between mental health and watching pornography.

## Methods

### Participants and procedures

Three surveys focusing on sexual behaviors and experiences of sexual abuse and sexual exploitation were conducted in Sweden among high school seniors (3rd year of high school) in 2004, 2009 and 2014 [29–31]. A set of core questions remained unchanged throughout the survey cycles to enable comparisons over time. Other questions were altered (deleted or added) to allow for the exploration of new societal trends, such as the development and use of the Internet and social media. The methodology shifted from paper-and-pencil format in 2004 and 2009 to a computerized survey in 2014. Some differences were also seen in the sample procedure (see below) over the years, but all three surveys aimed to gather a national probability-based, sample of Swedish high-school seniors aged 18 on average, by performing a stratified sampling procedure based on school size and educational programs.

According to data provided by Statistics Sweden (2006, 2014) [32, 33], approximately 90% of all 18-year-old adolescents were enrolled in Swedish high schools during this period. At the start of the initial survey in 2004, following the selection high schools included in the sample, the director of the entire school system in each participating community was asked to grant permission to present the research project to the principal of each high school. Once permission had been obtained from the principal of a school, consent was requested through oral and written information provided to the students. Students then gave informed consent to fill out the questionnaires. According to the Ethical Review Act of Sweden [34], parental consent is not required in Sweden if the respondent is older than 15 years of age.

The Human Research Ethics Committee, Medical Faculty, Lund University (2003, Dnr 938-02) and the Regional Ethical Review Board of Linköping (2009, Dnr 220-08 and 2014, Dnr 131-31) approved the studies. All students were given written information about the location and availability of counseling services.

#### The 2004 study

The sample consisted of Swedish high school seniors (3rd-year students). The guidelines of the Baltic Sea Regional Study on Adolescent Sexuality [35] were followed when selecting Stockholm (capital), Malmö (large port), as well as Luleå, Haparanda, and Falköping (smaller cities) for data collection. Students from surrounding villages and rural areas who commuted to the schools in these cities were included in the sample. All 3rd-year high school students in these cities were included in the initial group. At the time of the implementation of the study in 2003, a total of 10,751 high school seniors were registered at the selected schools, where 17 different national educational programs were offered. By applying a sampling procedure designed to ensure proportional representation of all programs and classes, 52.3% of the total number of students were selected, giving 4377 participants for the study. Thirty-eight questionnaires had to be excluded because they had not been completed correctly. The final number of participants was 4339 (2015 boys and 2324 girls), yielding a response rate of 77.2%. Seventy-three children did not answer the index question have you ever watched pornography, rendering 4266 adolescents (1967 boys and 2299 girls) with a mean age of 18.15 years (SD = 0.74) included in the analysis.

#### The 2009 study

The sampling frame consisted of all students in the previous year (second year) of high school at schools with at least 10 students. In total, there were 123,551 students at 754 schools in 2007. The sampling frame was stratified for number of students enrolled at each school (10–190, 191–360 and > 360 students) and educational program (20 programs). A random sample that included at least one program at 150 schools was selected (7700 students) and 119 schools chose to participate. Of the 5792 enrolled students at these schools, 3503 students participated in the study resulting in a response rate of 60.5%. Students were excluded from the sample if they provided unserious or incomplete responses (*n* = 5), if they did not report their sex (*n* = 38) or if they answered, ‘‘I do not fit within the division of male and female” (*n* = 28). Of the remaining participants, 149 did not answer the index question have you ever watched pornography, rendering 3283 students (1507 boys and 1776 girls) with a mean age of 18.26 years (SD = 0.60).

#### The 2014 study

A total of 13,903 senior year students from 261 out of 1215 high schools were selected for the study. Of the 261 schools selected, 238 were still providing the selected study programs in 2014. Schools that agreed to participate in the study amounted to 171, and of the 9773 students enrolled, 5873 agreed to participate in the study and completed the questionnaire. Note that an oversampling was made for the city of Stockholm to compare results to the county of Stockholm in a separate study, but since differences in the variables of interest among the samples were negligible, the additional sampling in Stockholm remained in the analysis. Thirty-four questionnaires were excluded due to unserious answers or a high amount of missing data, yielding a response rate of 59.7%. Participants were excluded if they did not report their sex (*n* = 2) or answered, ‘‘I do not fit within the division of male and female’’ (*n* = 54). Of the remaining participants, 105 did not answer the index question have you ever watched pornography, rendering 5678 students (2530 boys and 3148 girls) with a mean age of 17.97 years (SD = 0.63).

### Measures

At each time interval, participants were asked a set of questions about watching pornography as described in Table 1. The item “Have you ever watched pornography?” yielded a yes–no response alternative. There was no distinction made between unintentional and intentional watching. To measure frequency of watching pornography, participants were asked “How many times have you watched pornography during the last 12 months?” They could select one of the following multiple choices: “Once or twice a year”; “Sometime each month”; “Sometime each week”; and “More or less daily”. Deviant pornography was defined by the question “What sort of pornography have you been watching”? followed by three examples: Sex with violence or force (violent pornography), sex between adults and children (a child is a person younger than 15 years of age) and sex with animals (animal pornography).

**Table 1:** Ever watching and frequency of watching pornography in the three samples

	2004			2009			2014		
	Boys	Girls	*p* value	Boys	Girls	*p* value	Boys	Girls	*p* value
	*n* (%)	*n* (%)		*n* (%)	*n* (%)		*n* (%)	*n* (%)	
Ever watched pornography			< 0.001			< 0.001			< 0.001
No	48 (2.4)	546 (23.7)		109 (7.2)	613 (34.5)		229 (9.1)	1575 (50.0)	
Yes	1919 (97.6)	1753 (76.3)		1398 (92.8)	1163 (65.5)		2301 (90.9)	1573 (50.0)	
Frequency of watching			< 0.001			< 0.001			< 0.001
Never or once/twice a year	525 (28.7)	1199 (88.7)		291 (20.7)	1124 (84.4)		432 (17.3)	2456 (79.6)	
Never used	48 (2.6)	546 (40.4)		109 (7.8)	613 (46.0)		229 (9.2)	1575 (51.1)	
Once or twice a year	477 (26.1)	653 (48.3)		182 (12.9)	511 (38.4)		203 (8.1)	881 (28.6)	
Some time each month	563 (30.8)	117 (8.7)		385 (27.4)	159 (11.9)		468 (18.7)	424 (13.7)	
Weekly or daily	742 (40.5)	35 (2.6)		730 (51.9)	49 (3.7)		1601 (64.0)	204 (6.6)	
Some time each week	543 (29.7)	31 (2.3)		521 (37.1)	45 (3.4)		1006 (40.2)	185 (6.0)	
More or less daily	199 (10.9)	4 (0.3)		209 (14.9)	4 (0.3)		595 (23.8)	19 (0.6)	

Questions regarding participants' mental health varied to some extent over the three surveys. In the study from 2004, The Mental Health scale consisted of six questions slightly modified from the SCL-90 depressive scale [36, 37]. The six questions concerned problems and worries experienced during the preceding week (felt that everything was a struggle; had any trouble sleeping; felt unhappy; miserable, or depressed; felt a sense of hopelessness about the future; felt tied up or tense; and worried too much about things). Each question on the Mental Health Scale could be scored between a range of 1–4 (1 = does not correspond at all to 4 = corresponds very well), with the total score ranging from 6 to 24. The internal consistency, assessed using Cronbach’s alpha, was 0.84 in the 2004 sample [37]. A cut-off was set at the 80th percentile resulting in a cut-off point of > 19 points indicating poor mental health.

For the 2009 study, the Symptom Check List-25 (SCL- 25) was used [38, 39]. SCL-25 has been developed from SCL-90 [36] and measures occurrence of psychiatric symptoms, mainly depression and anxiety, during the previous week. The measure consists of 25 items with a 4-point scale ranging from 1 = not at all to 4 = extremely. A total score ranging from 25 to 100 can be calculated with high values indicating a high symptom score. Cronbach’s alpha was 0.93 in this study [40]. A cutoff was set at the 80th percentile with values > 53 indicating poor mental health. SCL-25 has been shown to have acceptable reliability and validity [38, 39].

The Trauma Symptom Checklist for Children (TSCC) [41, 42] was used in the 2014 study. The questionnaire includes 54 questions that can be divided into six clinical categories: anxiety, depression, post-traumatic stress, sexual concerns, dissociation, and anger. Response options are 0 = never, 1 = sometimes, 2 = often and 3 = almost all of the time. A total score ranging from 0 to 162 can be calculated with high values indicating a high symptom score. Cronbach’s alpha in the present sample was 0.95 for the full instrument [43]. A cut-off was set at 80th percentile with values > 44 indicating poor mental health. TSCC has been shown to have acceptable reliability and validity [41, 42]. Due to the modality of the TSCC scale, additional analyses were performed on the 80th percentile of the combined subscales for anxiety and depression.

For the multiple logistic regression analyses, a set of nine control variables identical in the three data set was used, shown in Table 2. Four of these were selected based on findings drawn from Peter and Valkenburg’s [1] 20-year review of pornography research which has shown that higher academic achievement (studying on a vocational or theoretical program, both yes or no), rule-breaking behavior, exemplified by truancy (2004 and 2009 the question was “Have you skipped school?” in 2014 the question was “Have you frequently skipped school?”, yes/no) and ever tried hashish/cannabis (yes/no), weak or troubled family relations represented by questions of low caring parents (“They cared about me”, yes/no on one or both parents) and high controlling parents (“Tried to control everything I did”, yes/no on one or both parents), and victimization (see sexual abuse below) are variables related to watching pornography. Five more control variables, used in earlier studies, and also identical in the three data sets, were used and these were living conditions (living with both parents, yes/no), economic status (employed mother, employed father, yes/no), immigrant status (born in Sweden, born outside Sweden, yes/no), early sexual debut (before the age of 14 years) and two questions concerning sexual victimization specifically (experienced all forms of sexual abuse and experienced penetrating sexual abuse (oral, anal or genital penetration, yes/no [22, 44–47].

**Table 2:** Health and descriptive data on participating boys, scoring high on respective measure on mental health in 2004, 2009, and 2014

	2004 (*n* = 1873–1963)			2009 (*n* = 1471–1520)			2014 (*n* = 2066–2264)		
	Mental Health scale, above 80th percentile			SCL-25, above 80th percentile			TSCC, above 80th percentile		
	No	Yes	*p* value	No	Yes	*p* value	No	Yes	*p* value
	*n* (%)	*n* (%)		*n* (%)	*n* (%)		*n* (%)	*n* (%)	
Living conditions			0.080			0.015			< 0.001
Living with both parents^1^	1262 (71.7)	126 (65.6)		986 (73.3)	106 (64.2)		1461 (74.6)	183 (60.0)	
Not living with both parents	499 (28.3)	66 (34.4)		360 (26.7)	59 (35.8)		497 (25.4)	122 (40.0)	
Economic status									
Employed mother	1387 (80.3)	125 (65.8)	< .001	1167 (87.1)	125 (77.2)	0.001	1731 (94.0)	249 (87.7)	< .001
Employed father	1412 (84.0)	135 (72.2)	< .001	1203 (91.4)	132 (85.2)	0.011	1766 (92.3)	254 (86.4)	0.001
Study program			0.504			0.005			0.012
Vocational	735 (41.8)	85 (44.3)		619 (45.7)	95 (57.2)		702 (35.8)	132 (43.3)	
Theoretical	1025 (58.2)	107 (55.7)		735 (54.3)	71 (42.8)		1257 (64.2)	173 (56.7)	
Immigrant status			< 0.001			0.023			0.551
Born in Sweden	1527 (86.3)	137 (70.6)		1265 (94.1)	145 (89.5)		1799 (91.8)	277 (90.8)	
Born outside of Sweden	242 (13.7)	57 (39.4)		79 (5.9)	17 (10.5)		160 (8.2)	28 (9.2)	
Rule-breaking									
Truancy^2^	1394 (82.4)	164 (90.1)	0.009	1066 (80.4)	148 (92.5)	< 0.001	332 (17.0)	105 (34.5)	< 0.001
Tried hashish/cannabis	441 (25.2)	70 (36.5)	0.001	296 (22.1)	62 (38.3)	< 0.001	465 (23.8)	101 (33.4)	< 0.001
Weak family									
Low caring parents	34 (1.9)	5 (2.6)	0.512	42 (3.1)	12 (7.4)	0.006	47 (2.4)	28 (9.2)	< 0.001
High controlling parents	63 (3.6)	24 (12.6)	< 0.001	139 (10.4)	45 (27.6)	< 0.001	222 (11.4)	95 (31.4)	< 0.001
Early sexual debut^3^	99 (8.1)	18 (12.6)	0.067	82 (8.7)	24 (19.7)	< 0.001	89 (7.4)	34 (16.4)	< 0.001
Sexual abuse^4^	378 (21.3)	73 (37.6)	< 0.001	68 (5.1)	26 (16.0)	< 0.001	123 (6.9)	54 (18.9)	< 0.001
Penetrating abuse	88 (5.0)	20 (10.3)	0.002	35 (2.6)	12 (7.4)	0.001	37 (2.1)	15 (5.3)	0.001
Ever watched pornography			0.771			0.009			0.065
No	43 (2.5)	4 (2.1)		87 (6.6)	19 (12.3)		178 (9.2)	18 (5.9)	
Yes	1706 (97.5)	185 (97.9)		1238 (93.4)	135 (87.7)		1767 (90.8)	285 (94.1)	
If yes: watched deviant pornography^5^	301 (17.6)	51 (27.6)	0.001	207 (16.7)	36 (26.7)	0.004	199 (11.3)	65 (22.8)	< 0.001
Frequency of watching			0.072			0.298			0.020
Some time each month	504 (30.9)	53 (30.5)		346 (28.0)	34 (22.8)		383 (19.9)	42 (13.9)	
Never or once/twice a year	475 (29.1)	38 (21.8)		248 (20.1)	36 (24.2)		328 (17.0)	46 (15.2)	
Weekly or daily	652 (40.0)	83 (47.7)		640 (51.9)	79 (53.0)		1215 (63.1)	214 (70.9)	

^1^Living with both parents include “alternating between mother and father”

^2^In 2004 and 2009 the question was “Have you skipped school?”, in 2014, the question was “Have you frequently skipped school?”

^3^Before the age of 14 years (only those who had had their sexual debut)

^4^The question about fondling was more strictly defined in 2009 and 2014 studies

^5^Included violent, animal and child pornography

In both the single and multiple logistic regression analyses, the frequency of watching pornography within the last year was divided into three categories: never or once/twice a year, some time each month and weekly or daily. This addresses the low frequency of responses in the two categories some time each week and more or less daily, in particular among girls. The variable deviant pornography was also created to encompass violent pornography, child sexual abuse material, and animal pornography.

### Statistical analysis

Descriptive data are presented as number (*n*) and percent (%). Initial analyses included bivariate analyses between sex and frequency of pornography watching in the three cohorts (2004, 2009, and 2014) using Pearson’s *Χ*^2^. In subsequent analyses, data were stratified by sex to account for different patterns of pornography watching, based on previous studies [1]. In these stratified analyses, Pearson’s *Χ*^2^ was used to analyze the bivariate relationship between the health status variables in 2004, 2009, and 2014 (each year analyzed separately) and socio-demographic background variables (living condition, parental employment, immigrant status, study program), behavioral variables (truancy, and use of cannabis), family variables (low caring parents, high controlling parents), sexual debut (before the age of 14 years) sexual abuse (ever abused, penetrating abuse) and frequency of watching pornography (ever watched, frequency of watching). In cases where cell count was below five, Fisher’s exact test was used instead of Pearson’s chi-square. Cases had partial missing values, number of missing values ranged between 1.1% and 7.9%. Missing data on the health variables were for boys and girls respectively, 2004; 2.2% and 2.0%, 2009; 4.6% and 2.5%, and for 2014; 2.7% and 7.9%. Similarly, missing data on ever having watched pornography were 2004; 2.4% and 1.1%, 2009; 5.5% and 3.4%, and 2014; 3.8% and 3.1% for boys and girls, respectively. Each of these cases were excluded from the analyses where this/these variable(s) were included in the analyses.

Single and multiple logistic regression models were performed, estimating the unadjusted and adjusted odds ratio of poor mental health for each independent variable. Subgroup analyses were performed on the group of adolescents who had watched pornography to evaluate whether deviant pornography increased the risk for mental health problems compared to other forms of pornography. The results are presented with unadjusted odds ratios (aOR), adjusted odds ratios (aOR) and effect sizes when significant aOR. Cohen’s d was used to calculate effect size with 0.20 0.50, and 0.80 for small, moderate, and large effect [48] As the multiple logistic regression models included a number of variables not reaching statistical significance, forward stepwise regression models (including the same variables) on the total population by year were performed. This was done in an attempt to verify the findings from the multiple logistic regression models by identifying the most important variables associated with poorer mental health.

Even if the focus is on the associations between adolescents watching pornography and poor mental health in the three surveys, we also chose to report the outcome of the included control variables in the result section and tables.

In 2004, the study population was not evaluated with respect to non-response/participation. However, in 2009 and 2014, this was executed, and it was found that some schools were underrepresented (e.g., schools from urban municipalities). To account for the skewness, weights were calculated to adjust the estimates accordingly. However, the effect on the estimates were negligible and thus it was decided to not include this correction in the models.

All analyses were performed using IBM SPSS version 26 (IBM Inc., Armonk, NY, USA). Statistical significance was defined as *p* < 0.001 (two-sided).

## Results

### Watching pornography

Both the prevalence of ever having watched pornography (lifetime) and the frequency of watching pornography have been presented in a recent paper [8]. This study found that the number of boys having ever watched pornography significantly decreased from 98 (2004) to 93% (2009) and finally to 91% (2014), while the prevalence for girls fell from 76 (2004), to 66% (2009) and to 50% (Table 1). During the same time, the proportion of students who reported watching pornography weekly or daily within the last year increased—for boys from 41 (2004) to 64% (2014) and for girls from 3 (2004) to 7% (2014), Table 1. Compared to girls, boys were significantly more likely to ever have watched pornography and watch pornography more frequently across all surveys (*p* < 0.001), Table 1.

### Boys

#### Bivariate analyses

The bivariate analyses showed no significant association between mental health and ever having watched pornography in 2004. Meanwhile, a positive association with poorer mental health in 2014 and ever watching pornography increased the odds for poor mental health, uOR 1.75 (1.09–2.81), but decreased the odds for poor mental health in 2009 among boys, uOR 0.50 (0.30–0.85), Tables 2, 3. A significant association between the frequency of watching pornography (weekly or daily) and poor mental health was found in 2014 only, uOR 1.58 (1.14–2.18). Having ever watched deviant pornography increased the odds of poor mental health over all three surveys, Tables 2, 3.

**Table 3:** Logistic regression, health 2004, 2009 and 2014, boys

	2004 Mental Health scale, above 80th percentile		2009 SCL-25, above 80th percentile		2014 TSCC, above 80th percentile	
	uOR (95% CI)	aOR (95% CI)	uOR (95% CI)	aOR (95% CI)	uOR (95% CI)	aOR (95% CI)
Living conditions						
Living with both^1^	Reference	Reference	Reference	Reference	Reference	Reference
Not living with both	1.32 (0.97–1.82)	0.98 (0.62–1.59)	1.52 (1.09–2.14)	1.14 (0.70–1.86)	1.86 (1.48–2.35)	1.30 (0.89–1.91)
Economic status						
Unemployed mother, yes^2^	2.12 (1.53–2.92)	1.80 (1.11–2.93)*	2.00 (1.34–2.98)	2.03 (1.15–3.59)*	1.96 (1.33–2.87)	2.04 (1.11–3.73)*
Unemployed father, yes^2^	2.02 (1.43–2.86)	1.43 (0.84–2.42)	1.86 (1.14–3.01)	1.79 (0.90–3.57)	1.72 (1.22–2.43)	1.50 (0.86–2.63)
Study program						
Vocational	Reference	Reference	Reference	Reference	Reference	Reference
Theoretical	0.90 (0.67–1.22)	1.08 (0.70–1.66)	0.63 (0.46–0.87)	0.85 (0.54–1.34)	0.79 (0.63–0.99)	0.99 (0.69–1.43)
Immigrant status						
Born in Sweden	Reference	Reference	Reference	Reference	Reference	Reference
Born outside of Sweden	2.62 (1.87–3.68)	2.85 (1.75–4.64)**	1.88 (1.08–3.26)	0.76 (0.30–1.88)	1.01 (0.68–1.48)	0.72 (0.36–1.43)
Rule-breaking						
Truancy^3^, yes^2^	1.94 (1.17–3.21)	1.24 (0.62–2.48)	3.01 (1.64–5.50)	2.49 (0.87–7.10)	2.58 (2.03–3.29)	1.82 (1.24–2.68)*
Tried hashish/cannabis, yes^2^	1.70 (1.24–2.32)	1.39 (0.89–2.17)	2.19 (1.55–3.08)	1.76 (1.09–2.83)**	1.62 (1.28–2.06)	1.06 (0.73–1.54)
Weak family						
Low caring parents, yes^2^	1.37 (0.53–3.55)	1.53 (0.50–4.63)	2.45 (1.26–4.76)	1.01 (0.34–3.03)	3.60 (2.30–5.64)	3.91 (1.86–8.22)**
High controlling parents, yes^2^	3.88 (2.36–6.38)	3.10 (1.54–6.22)**	3.28 (2.23–4.83)	2.01 (1.11–3.63)*	3.28 (2.52–4.26)	2.67 (1.78–4.01)**
Early sexual debut^4^, yes^2^	1.64 (0.96–2.80)	0.87 (0.42–1.79)	2.56 (1.55–4.22)	1.86 (1.00–3.46)	2.32 (1.59–3.40)	2.03 (1.20–3.41)*
Sexual abuse^5^, yes^2^	2.23 (1.63–3.05)	1.75 (1.09–2.82)*	3.56 (2.19–5.78)	0.92 (0.25–3.32)	2.84 (2.06–3.92)	2.23 (1.21–3.40)*
Penetrating abuse, yes^2^	2.20 (1.32–3.67)	0.75 (0.33–1.71)	2.97 (1.51–5.85)	2.65 (0.60–11.70)**	2.12 (1.20–3.73)	1.10 (0.44–2.71)
Ever watched pornography						
No	Reference	Reference	Reference	Reference	Reference	Reference
Yes	1.17 (0.41–3.28)	1.02 (0.19–5.45)	0.50 (0.30–0.85)	0.35 (0.14–0.92)	1.75 (1.09–2.81)	1.82 (0.69–4.81)
If yes: watched deviant pornography^6^	1.78 (1.26–2.51)	1.74 (1.08–2.81)*	1.81 (1.20–2.73)	1.60 (0.91–2.80)	2.26 (1.69–3.04)	1.73 (1.13–2.65)*
Frequency of watching						
Some time each month	Reference	Reference	Reference	Reference	Reference	Reference
Never or once/twice a year	0.76 (0.49–1.18)	0.86 (0.47–1.49)	1.48 (0.90–2.43)	1.08 (0.51–2.29)	1.19 (0.79–1.80)	2.64 (1.24–5.60)**
Weekly or daily	1.21 (0.84–1.74)	1.04 (0.64–1.71)	1.26 (0.82–1.92)	0.86 (0.50–1.46)	1.58 (1.14–2.18)	2.34 (1.37–3.97)*

^1^Living with both parents include “alternating between mother and father”

^2^No used as reference

^3^In 2004 and 2009, the question was “Have you skipped school?”, in 2014 the question was “Have you frequently skipped school?”

^4^Before the age of 14 years (only those who had had their sexual debut),

^5^The question about fondling was more strictly defined in 2009 and 2014 studies

^6^Included violent, animal and child pornography

*Effect size = 0.20

**Effect size = 0.50

***Effect size 0.80

Most of the control variables increased the odds for poor mental health. These include parental unemployment (all years), not living with both parents and respondent born outside Sweden (2004 and 2009), rule breaking (all years), low caring parents (2009 and 2014), high controlling parents (all years), early sexual debut (2009 and 2014) and sexual abuse/penetrating sexual abuse (all years). Studying in a theoretical school program decreased the ORs both in 2009 and 2014, Tables 2, 3.

### Boys

#### Multiple analyses

In the multiple logistic regression outlined in Table 3, ever having watched pornography decreased the odds for poor mental health in 2009 aOR 0.35 (0.14–0.92), while low frequency of watching pornography (never or once/twice a year) and high frequency (weekly or daily) increased the odds for poor mental health in 2014, aOR 2.64 (1.24–5.60) and aOR 2.34 (1.37–3.97) respectively, Table 3. Ever having watched deviant pornography increased the odds for poor mental health both in 2004 aOR1.74 (1.08–2.81) and in 2014, aOR 1.73 (1.13–2.65) according to the instrument used, Table 3.

For the control variables, having an unemployed mother and having high controlling parents increased the odds for poor mental health in all surveys while sexual abuse increased the ORs in 2004 aOR1.75 (1.09–2.82) and 2014 aOR 2.23 (1.21–3.40). Several control variables only increased the ORs for poor mental health in one of the surveys, such as being born outside Sweden in the 2004 survey aOR 2.85 (1.75–4.64), truancy aOR 1.82 (1.24–2.68) and trying hashish/cannabis in the 2009 survey aOR 1.76 (1.09–2.83), or low caring parents aOR 3.91 (1.86–8.22) and early sexual debut in the 2014 survey aOR 2.03 (1.20–3.41). In terms of effect sizes, the overall most important variables across the years were having an unemployed mother (effect size = 0.32–0.39) and controlling parents (effect size = 0.38–0.62).

The stepwise forward logistic regression identified several variables of importance for poor mental health among boys, Table 6. These were having an unemployed mother (all years) and having high controlling parents (all years), early sexual debut (2009 and 2014), sexual abuse (2004) and penetrative sexual abuse (2009), respondent born outside Sweden (2004), truancy (2014), tried hashish/cannabis (2009), and having low caring parents (2014). Both low and high frequency of watching pornography in the preceding year were associated with poor mental health in the survey from 2014.

Compared to the analyses on the total TSCC scale, the additional analyses on the combined subscales for anxiety and depression yielded diverging findings. Statistical significances on having an employed mother, truancy, early sexual debut, sexual abuse and having watched deviant pornography vanished among boys. Also, among boys, the independent variables with the highest effect size were having low caring parents, controlling parents, and frequency of watching (all effect sizes were above 0.65).

### Girls

#### Bivariate analyses

In the bivariate analyses for girls, ever having watched pornography significantly increased poor mental health in both 2004 uOR 1.54 (1.18–1.99) and 2014 uOR 2.37 (2.01–2.79), Tables 4, 5. Low frequency of watching pornography (never or once/twice a year) decreased poor mental health both in 2009 uOR 0.67 (0.47–0.96) and in 2014 uOR 0.53 (0.43–0.66), while high frequency of watching pornography (weekly or daily) increased the OR for poor mental health among girls in 2014 uOR 1.54 (1.10–2.17). Ever having watched deviant pornography also increased the ORs for poor mental health both in 2009 uOR 1.57 (1.04–2.37) and in 2014 uOR 2.12 (1.51–3.04).

**Table 4:** Health and descriptive data on participating girls, scoring high on respective measure on mental health in 2004, 2009, and 2014

	2004 (*n* = 2159–2273)			2009 (*n* = 1719–1792)			2014 (*n* = 2713–2931)		
	Mental Health scale, above 80th percentile			SCL-25, above 80th percentile			TSCC, above 80th percentile		
	No	Yes	*p* value	No	Yes	*p* value	No	Yes	*p* value
	*n* (%)	*n* (%)		*n* (%)	*n* (%)		*n* (%)	*n* (%)	
Living conditions			0.083			< 0.001			< 0.001
Living with both^1^	1232 (68.1)	289 (63.8)		885 (68.2)	255 (55.2)		1640 (74.2)	451 (62.6)	
Not living with both	578 (31.9)	164 (36.2)		412 (31.8)	207 (44.8)		569 (25.8)	269 (37.4)	
Economic status									
Employed mother	1470 (81.8)	331 (74.7)	0.001	1130 (86.1)	363 (77.7)	< 0.001	1951 (93.8)	621 (93.4)	0.702
Employed father	1440 (82.4)	332 (75.5)	0.001	1130 (88.7)	376 (84.5)	0.021	1959 (90.9)	606 (87.8)	0.020
Study program			0.784			0.005			0.001
Vocational	662 (36.7)	163 (36.0)		452 (34.2)	195 (41.5)		550 (24.9)	225 (31.2)	
Theoretical	1143 (63.3)	290 (64.0)		870 (65.8)	275 (58.5)		1660 (75.1)	496 (68.8)	
Immigrant status			< 0.001			0.744			0.951
Born in Sweden	1573 (86.6)	363 (79.6)		1215 (92.7)	434 (93.1)		2046 (92.6)	667 (92.5)	
Born outside of Sweden	244 (13.4)	93 (20.4)		96 (7.3)	32 (6.9)		164 (7.4)	54 (7.5)	
Rule-breaking									
Truancy^2^	1423 (82.2)	373 (87.1)	0.014	1034 (79.7)	399 (87.7)	< 0.001	351 (15.9)	296 (41.1)	< 0.001
Tried hashish/cannabis	354 (19.7)	127 (28.0)	< 0.001	163 (12.4)	105 (22.7)	< 0.001	312 (14.2)	179 (25.0)	< 0.001
Weak family									
Low caring parents	18 (1.0)	19 (4.1)	< 0.001	29 (2.2)	41 (8.8)	< 0.001	79 (3.6)	50 (7.0)	< 0.001
High controlling parents	70 (3.9)	46 (10.2)	< 0.001	145 (11.0)	81 (17.4)	< 0.001	172 (7.8)	144 (20.0)	< 0.001
Early sexual debut^3^	82 (6.3)	28 (8.4)	0.186	97 (10.1)	45 (12.1)	0.287	87 (6.1)	57 (10.5)	0.001
Sexual abuse^4^	1133 (62.2)	341 (74.6)	< 0.001	267 (20.3)	182 (39.1)	< 0.001	454 (21.6)	351 (50.7)	< 0.001
Penetrating abuse	203 (11.1)	99 (21.7)	< 0.001	89 (6.8)	93 (20.0)	< 0.001	117 (5.6)	130 (18.8)	< 0.001
Ever watched pornography			0.001			0.106			< 0.001
No	455 (25.2)	82 (18.0)		460 (35.4)	141 (31.2)		1213 (55.0)	245 (34.2)	
Yes	1352 (74.8)	374 (82.0)		840 (64.6)	311 (68.8)		991 (45.0)	472 (65.8)	
If yes: watched deviant pornography^5^	95 (7.0)	37 (9.9)	0.065	72 (8.6)	40 (12.9)	0.029	65 (6.6)	62 (13.1)	< 0.001
Frequency of watching			0.282			0.080			< 0.001
Some time each month	91 (8.4)	26 (10.5)		105 (10.9)	53 (15.3)		268 (12.4)	132 (18.8)	
Never or once/twice a year	970 (89.2)	212 (85.8)		827 (85.5)	279 (80.6)		1797 (83.0)	483 (68.8)	
Weekly or daily	26 (2.4)	9 (3.6)		35 (3.6)	14 (4.0)		99 (4.6)	87 (12.4)	

^1^Living with both parents include “alternating between mother and father”

^2^In 2004 and 2009 the question was “Have you skipped school?”, in 2014, the question was “Have you frequently skipped school?”

^3^Before the age of 14 years (only those who had had their sexual debut)

^4^The question about fondling was more strictly defined in 2009 and 2014 studies

^5^Included violent, animal and child pornography

**Table 5:** Logistic regression, health 2004, 2009 and 2014, girls

	2004 6 questions, above 80th percentile		2009 SCL-25, above 80th percentile		2014 TSCC, above 80th percentile	
	uOR (95% CI)	aOR (95% CI)	uOR (95% CI)	aOR (95% CI)	uOR (95% CI)	aOR (95% CI)
Living conditions						
Living with both^1^	Reference	Reference	Reference	Reference	Reference	Reference
Not living with both	1.21 (0.98–1.50)	0.69 (0.47–1.02)	1.74 (1.40–2.17)	1.67 (1.20–2.34)*	1.72 (1.45–2.03)	1.16 (0.90–1.51)
Economic status						
Unemployed mother, yes^2^	1.53 (1.19–1.95)	1.76 (1.13–2.72)*	1.77 (1.35–2.31)	1.35 (0.86–2.10)	1.02 (0.73–1.42)	1.09 (0.65–1.83)
Unemployed father, yes^2^	1.52 (1.18–1.95)	1.48 (0.92–2.38)	1.44 (1.06–1.96)	1.03 (0.63–1.68)	1.31 (1.01–1.69)	1.03 (0.68–1.56)
Study program						
Vocational	Reference	Reference	Reference	Reference	Reference	Reference
Theoretical	1.03 (0.83–1.28)	0.97 (0.67–1.40)	0.73 (0.59–0.91)	0.82 (0.59–1.13)	0.73 (0.62–0.87)	0.78 (0.60–1.01)
Immigrant status						
Born in Sweden	Reference	Reference	Reference	Reference	Reference	Reference
Born outside of Sweden	1.65 (1.27–2.15)	1.10 (0.64–1.91)	0.93 (0.62–1.41)	0.68 (0.31–1.46)	1.05 (0.79–1.40)	0.92 (0.54–1.58)
Rule-breaking						
Truancy^3^, yes^2^	1.47 (1.08–2.00)	1.51 (0.78–2.93)	1.82 (1.33–2.48)	2.80 (1.57–5.00)**	3.65 (3.06–4.35)	2.39 (1.84–3.11)*
Tried hashish/cannabis, yes^2^	1.59 (1.26–2.01)	1.28 (0.85–1.93)	2.08 (1.58–2.73)	1.49 (1.01–2.20)*	2.04 (1.68–2.48)	1.07 (0.80–1.42)
Weak family						
Low caring parents, yes^2^	4.39 (2.29–8.44)	5.39 (1.57–18.48)***	4.27 (2.62–6.96)	1.76 (0.82–3.79)	2.07 (1.48–2.90)	0.96 (0.51–1.81)
High controlling parents, yes^2^	2.83 (1.92–4.16)	1.31 (0.64–2.69)	1.70 (1.26–2.28)	1.45 (0.91–2.30)	3.00 (2.40–3.75)	2.05 (1.45–2.90)*
Early sexual debut^4^, yes^2^	1.35 (0.86–2.11)	0.95 (0.47–1.92)	1.23 (0.84–1.78)	0.56 (0.32–0.99)	1.71 (1.23–2.38)	0.77 (0.49–1.22)
Sexual abuse^5^, yes^2^	1.78 (1.42–2.25)	2.03 (1.24–3.32)*	2.51 (2.00–3.16)	1.27 (0.83–1.93)	3.75 (3.16–4.46)	2.62 (1.99–3.43)**
Penetrating abuse, yes^2^	2.20 (1.69–2.88)	1.68 (1.08–2.64)*	3.43 (2.51–4.69)	2.12 (1.26–3.57)**	3.76 (2.92–4.85)	1.52 (1.06–2.18)*
Ever watched pornography						
No	Reference	Reference	Reference	Reference	Reference	Reference
Yes	1.54 (1.18–1.99)	1.11 (0.71–1.73)	1.21 (0.96–1.52)	0.92 (0.64–1.34)	2.37 (2.01–2.79)	1.46 (1.10–1.93)*
If yes: watched deviant pornography^6^	1.45 (0.98–2.16)	1.57 (0.86–2.86)	1.57 (1.04–2.37)	1.41 (0.80–2.46)	2.15 (1.51–3.04)	1.88 (1.15–3.08)*
Frequency of watching						
Some time each month	Reference	Reference	Reference	Reference	Reference	Reference
Never or once/twice a year	0.76 (0.48–1.21)	0.84 (0.48–1.45)	0.67 (0.47–0.96)	0.90 (0.57–1.42)	0.53 (0.43–0.66)	0.81 (0.58–1.13)
Weekly or daily	1.21 (0.50–2.90)	1.09 (0.37–3.19)	0.79 (0.39–1.60)	1.20 (0.53–2.71)	1.54 (1.10–2.17)	1.47 (0.92–2.34)

^1^Living with both parents include “alternating between mother and father”

^2^No used as reference

^3^In 2004 and 2009, the question was “Have you skipped school?”, in 2014, the question was “Have you frequently skipped school?”

^4^Before the age of 14 years (only those who had had their sexual debut),

^5^The question about fondling was more strictly defined in 2009 and 2014 studies

^6^Included violent, animal and child pornography

*Effect size = 0.20

**Effect size = 0.50

***Effect size =0.80

The studied control variables showed increased uORs for not living with both parents (2009 and 2014), unemployed parents (2004 and 2009), a weak family (all years), rule-breaking behavior (all years) and sexual abuse/penetrative sexual abuse (all years). Being born outside Sweden increased the OR 2004 and early sexual debut in 2014. Similar to the boys, studying a theoretical school program decreased the uORs for poor mental health both in 2009 and 2014, Tables 4, 5.

### Girls

#### Multiple analyses

In the multiple logistic regression models, only ever watching pornography and watching deviant pornography increased the aORs for poor mental health in 2014, aOR 1.46 (1.10–1.93) and aOR 1.88 (1.15–3.08) respectively.

Table 5 shows that among the background variables controlled for, sexual abuse increased the aORs in two of the survey years, 2004 aOR 2.03 (1.24–3.32) and 2014 aOR 2.62 (1.99–3.43) while penetrative sexual abuse increased the ORs in all three survey years, 2004 aOR 1.68 (1.08–2.64), 2009 aOR 2.12 (1.26–3.57) and 2014 aOR 1.52 (1.06–2.18). Not living with both parents increased the aOR for poor mental health in 2009 aOR 1.67 (1.20–2.34) and having an unemployed mother increased the aOR in 2004 1.76 (1.13–2.72). Having low caring parents in 2004 and having high controlling parents in 2014 increased the aORs for poor mental health, aOR 5.39 (1.57–18.48) and aOR 2.05 (1.45–2.90) respectively. Rule-breaking behavior such as truancy increased the aORs for poor mental health in 2009 aOR 2.80 (1.57–5.00) and 2014 aOR 2.39 (1.84–3.11) and tried hashish/cannabis increased the aOR in 2009 aOR 1.49 (1.01–2.20). Early sexual debut decreased the aOR for poor mental health in 2009 aOR 0.56 (0.32–0.99). In evaluating the effect sizes, truancy (effect size = 0.48–0.57) and sexual abuse (effect size = 0.39–0.53), the variables found to have the greatest relative impact on the mental health.

Similar to the analysis among the boys, the stepwise forward logistic regression identified several variables of importance for the association with poor mental health among girls, Table 6. These were penetrative sexual abuse (all years), sexual abuse (2004 and 2014), truancy (2009 and 2014), not living with both parents (2009), having an unemployed mother (2004), having non-caring parents (2004) and controlling parents (2014). However, this model also found that ever having watched pornography was associated with a lower rate of poor mental health among girls in 2004.

**Table 6:** Stepwise regression models on mental health in 2004, 2009, and 2010 stratified by boys and girls

	2004		2009		2014	
	Boys	Girls	Boys	Girls	Boys	Girls
Living conditions						
Living with both^1^	–	–	–	Reference	–	–
Not living with both	–	–	–	1.86 (1.36–2.54)	–	–
Economic status						
Unemployed mother	1.85 (1.17–2.92)	1.92 (1.27–2.90)	2.31 (1.35–3.93)	-	2.05 (1.15–3.65)	-
Unemployed father	–	–	–	–	–	–
Study program						
Vocational	–	–	–	–	–	–
Theoretical	–	–	–	–	–	–
Immigrant status						
Born in Sweden	Reference	–	–	–	–	–
Born outside of Sweden	3.05 (1.89–4.93)	–	–	–	–	–
Rule-breaking						
Truancy^2^				2.93 (1.66–5.18)	1.93 (1.32–2.80)	2.50 (1.94–3.21)
Tried hashish/cannabis			1.87 (1.17–2.97)			
Weak family						
Low caring parents	–	5.38 (1.61–17.91)	–	–	4.26 (2.04–8.87)	–
High controlling parents	2.80 (1.43–5.51)	–	2.04 (1.14–3.62)	–	2.60 (1.74–3.88)	2.08 (1.48–2.94)
Early sexual debut^3^	–	–	2.03 (1.11–3.73)	–	2.04 (1.22–3.41)	–
Sexual abuse^4^	1.67 (1.09–2.56)	2.14 (1.33–3.47)	–	–	–	2.60 (1.98–3.40)
Penetrating abuse	–	1.76 (1.15–2.69)	2.68 (1.17–6.15)	2.55 (1.71–3.79)	–	1.53 (1.07–2.19)
Ever watched pornography						
No	–	Reference	–	–	–	–
Yes	–	0.32 (0.15–0.69)	–	–	–	–
Frequency of watching						
Some time each month	–	–	–	–	Reference	Reference^5^
Never or once/twice a year	–	–	–	–	2.03 (1.05–3.93)	0.82 (0.59–1.14)
Weekly or daily	–	–	–	–	2.24 (1.33–3.77)	1.46 (0.92–2.32)

^1^Living with both parents include “alternating between mother and father”

^2^In 2004 and 2009, the question was “Have you skipped school?”, in 2014, the question was “Have you frequently skipped school?”

^3^Before the age of 14 (only those who had had their sexual debut)

^4^The question about fondling was more strictly defined in 2009 and 2014 studies,

^5^The overall *p* value for this variable is 0025 and therefore included in the model (even though the presented confidence intervals contain 1.0

In the analyses on the reduced TSCC scale, where only the sub-scales of anxiety and depression were included, the statistically significant findings of increased odds ratios for penetrating sexual abuse and having watched pornography disappeared, whereas watching pornography daily–weekly was now found to increase the odds ratio for symptoms of anxiety and depression. The greatest effect sizes among girls were found on the variables truancy (0.37), sexual abuse (0.48), and controlling parents (0.39).

## Discussion

Using three school-based surveys, this study contributes to the knowledge of young people´s watching pornography and associations with poor mental health. It is one of the first studies using three different surveys using identical questions about watching pornography (ever and frequency last year) and a set of identical controlling background variables. There are four main findings derived from the study:

First, as reported in an earlier study [8], while there was an overall decrease in ever having watched pornography (lifetime) during the years surveyed, especially for girls, there was an increase in the proportion of adolescents who watch pornography frequently, especially among boys, where 64% watched pornography on a weekly or daily basis in the 2014 survey. These findings correspond with a recent study where men aged 16–24 watched pornography at least 1–2 times a week [49]. As discussed in the earlier 2021 study [8], the decreasing trend of ever having watched pornography could be explained by a growing group of adolescents who actively avoid watching pornography. It could also stem from varying definitions of pornography over time, where the normalization of sexual images in society may present challenges for adolescents when distinguishing pornographic content from other types of sexualized media they encounter online. Meanwhile, the growing proportion of boys watching pornography frequently over time is best explained by continuous technological advancements, where internet and mobile technologies allow for uninterrupted digital access, effectively reducing the effort required to access pornography.

Second, the repeated cross-sectional surveys did not find any consistent associations across years between poor mental health and ever having watched pornography or the frequency of watching pornography when controlling for background factors. In one survey cycle (2014), ever having watched pornography increased the aOR for poor mental health among girls, while in another, it decreased the aOR for poor mental health concerning boys (2009). As for the association between the frequency of watching pornography during the last year, a U-shaped pattern was observed in one of the surveys concerning boys, with an increased risk for poor mental health for both low frequent watchers (never or once/twice a year) and high frequent watchers (weekly or daily) compared to more moderate watchers (some time each month). This U-shaped relationship was also found with regard to the intensity of internet use and adolescent health (depressive scores) in a Swiss study with 7211 adolescents aged 16–20 years [50]. This suggests that high-consumers and low-consumers of the internet and online pornography both differ from mainstream consumers in terms of health.

Third, having watched deviant pornography, defined as pornography showing violence, children and/or animals, was significantly associated with poor mental health in five out of six bivariate analyses but only in three of the multivariate analyses (boys in two and girls in one of the surveys). This finding partly supports the notion that the actual content in the pornography watched has implications when it comes to poor psychosocial health, a finding in line with a study among Italian adolescents [51]. Whether exposure to deviant pornography contributes to poor mental health, whether poor mental health is a predictor for watching deviant pornography, or some combination of the two, remains unclear.

Overall, these findings suggest that watching pornography when growing up (lifetime) or in late adolescence (last year) may have a limited impact on poor mental health. These results are similar to findings by Kohut & Štulhofer and Štulhofer, Tafo and Kohut [26, 27], who found that watching pornography in middle to late adolescence did not contribute to adverse psychological well-being measured by self-esteem and anxiety/depression.

Fourth, several background variables were more consistently and more strongly associated to reported poor mental health among adolescents. The most evident were related either to family circumstances or to traumatic experiences like different forms of sexual abuse. This is not surprising, as previous research has shown that socioeconomic stress, such as parental unemployment or the perception of parental unemployment [52, 53], as well as parenting styles, such as overprotection/control, especially when combined with low care [54], are associated with poor mental health among children. It is also well established that exposure to traumatic experiences, especially child sexual abuse, is connected to poor mental health during childhood [55, 56], and has long-term term consequences [57]. Furthermore, the association found between early sexual debut and poor mental health among boys is consistent with results from other studies that have found that early sexual debut is associated with poor mental health among both boys and girls [58, 59]. The association between truancy and poor mental health among girls has also been observed in earlier research [60]. While these results could indicate that the relationship between pornography and mental health may be better explained by underlying confounding variables, the variables could very well be predictors, mediators, or moderators of the relationship between pornography and mental health. More research is thus needed to tease out this interconnected and complex relationships.

## Study strengths, limitations, and future directions

This study has several strengths. First, we used three different surveys to strengthen the generalizability of the results. Specifically, the identical index questions about watching pornography and background variables used throughout the survey cycles improve the validity of the results. Second, the use of nearly identical sampling procedures and including the same age group in three large samples is an additional strength of this study.

Using different questionnaires for measuring mental health in the three surveys could both be seen as both a strength and a limitation. If there had been a really robust connection between porn watching and mental health, it should have been shown no matter what questionnaire used. The selection of questionnaires was made at different points in time, and they were selected for the purposes of each of the surveys, and not explicitly for this study. The differences in the number of questions and profile between the three mental health questionnaires used and the lack of clinical cut-offs could jeopardize the validity of the results. The rationale behind not using clinical cut-offs was that mental health questionnaire used in the 2004 survey does not have a clinical cut-off. However, to minimize this risk, we used a common cut-off for the questionnaires.

The ambition to use three surveys to identify patterns of associations between poor mental health and the watching pornography cannot obscure the fact that each study had a cross-sectional design with the limitation of reporting associations, rather than explaining causation. Another shortcoming was that deviant pornography was restricted to three forms of pornography (depicting violence, children and/or animals). A greater diversity of types of pornography watched by adolescents could perhaps have contributed to a more diversified picture of the associations. Neither did we differentiate between unintentional and intentional watching. We were also limited to the background variables that were common in the three surveys, which is why other background variables, such as physical abuse, could not be studied. Regarding the operationalization of the frequency of watching pornography, a continuous scale could have contributed to a more refined measure of pornography watching, but on the other hand, our categorization is similar to another recent published study (49).

We did not directly assess possible associations between the dynamics of pornography watching (i.e., the rate of change in watching pornography over the three surveys) and the indicators of psychological well-being. Finally, we are well aware that the data from the last study are seven years old and patterns of use and for example the development of artificial intelligence algorithms might change the area and push consumers toward deviant pornography. Hopefully, a just-finished data collection from 2020 to 2021 can update the patterns of pornography use among adolescents.

Most previous researches focusing on young people’s mental health and watching pornography have not controlled for such a diverse set of background variables as was used in this study. The number of controls could be seen as both a strength and a limitation. Controlling for too many background variables runs the risk of obscuring connections between studied phenomena [61]. This can be discussed and must always be considered when studying associations between two variables, but our opinion is that bivariate associations should be supplemented with analyses where other significant variables are controlled for as in this study. Another limitation of this study is the focus on mental health without including sexual health or gender stereotypes that have previously been found to be related to adolescent pornography watching [1, 62]. These questions were not possible to include since they were not identical across the three survey years. Furthermore, self-reports at the age of 18 cover growing up but not mental health issues that have their basis during this period, but have not yet manifested themselves.

To further understand the role of potential mediators, moderators, and predictors in the relationship between pornography and mental health, the field would benefit from theoretically driven, longitudinal studies that can help to tease out the mechanisms driving particular outcomes. Furthermore, watching pornography through the privacy and convenience of a smartphone could hypothetically affect young people in different ways than accessing pornography on a computer or in a magazine. With this in mind, we recommend that future studies in this field consider the specific modalities (i.e., smartphone, laptop) by which young people consume pornography, as previous research has found that this can have an impact on the effects of pornography exposure [63].

## Conclusion

When studying the consequences of watching pornography and its impact on young people’s mental health, it is important to keep in mind that there are multiple and often intricate associations with a variety of life circumstances that affect the young person's health. Over the course of the three survey cycles, we found that background variables, such as family circumstances, sexual abuse, truancy, and early sexual debut, were more consistently linked to mental illness than watching pornography. However, we found that that watching deviant pornography was more often associated with poor mental health. This emphasizes that the adult world, and not least professionals in school, social services and child and adolescent psychiatry need to pay attention to the child’s entire life situation, especially traumatic experiences, such as child abuse and neglect, when considering adolescent’s pornography watching and mental health.

## Supplementary Information

Below is the link to the electronic supplementary material.

Supplementary file1 (DOCX 24 KB)
