# Supplementary material for: Associations between adolescents watching pornography and poor mental health in three Swedish surveys
Source: Eur Child Adolesc Psychiatry. 2022 May 7;32(9):1765–80. doi: 10.1007/s00787-022-01992-x (PMC10460300; doi:10.1007/s00787-022-01992-x)
Supplement: Supplementary file 1 — Supplementary file1 (DOCX 24 KB) [file 787_2022_1992_MOESM1_ESM.docx]

**S1.** Supplementary information on the crosstabulation as well as unadjusted and adjusted logistic regression models of the combined outcome based on

sTSCC anxiety and depression scales

|  | Boys | | | Girls | | | Boys | | Girls | |
| --- | --- | --- | --- | --- | --- | --- | --- | --- | --- | --- |
|  | No | Yes |  | No | Yes |  |  |  |  |  |
|  | n (%) | n (%) | p-value | n (%) | n (%) | p-value | uOR | aOR | uOR | aOR |
| Living conditions |  |  | <0.001 |  |  | <0.001 |  |  |  |  |
| Living with both^1^ | 1526 (73.8) | 118 (60.8) |  | 1553 (73.9) | 538 (65.0) |  | *Reference* | *Reference* | *Reference* | *Reference* |
| Not living with both | 543 (26.2) | 76 (39.2) |  | 548 (26.1) | 290 (35.0) |  | 1.81 (1.34-2.45) | 1.31 (0.82-2.09) | 1.53 (1.28-1.82) | 1.17 (0.91-1.51) |
| Economic status |  |  |  |  |  |  |  |  |  |  |
| Employed mother | 1821 (93.6) | 159 (87.8) | 0.003 | 1851 (93.2) | 721 (95.1) | 0.058 | 2.03 (1.26-3.29) | 1.14 (0.50-2.58) | 0.70 (4.81-1.02) | 0.82 (0.49-1.38) |
| Employed father | 1854 (91.7) | 166 (88.8) | 0.164 | 1854 (90.5) | 711 (89.2) | 0.306 | 1.40 (0.87-2.27) | 0.71 (0.32-1.61) | 1.15 (0.88-1.50) | 0.96 (0.84-1.44) |
| Study program |  |  | 0.811 |  |  | 0.142 |  |  |  |  |
| Vocational | 761 (36.8) | 73 (37.6) |  | 540 (25.7) | 235 (28.3) |  | *Reference* | *Reference* | *Reference* | *Reference* |
| Theoretical | 1309 (63.2) | 121 (62.4) |  | 1562 (74.3) | 594 (71.7) |  | 0.96 (0.71-1.31) | 1.36 (0.86-2.16) | 0.87 (0.73-1.05) | 0.90 (0.70-1.17) |
| Immigrant status |  |  | 0.183 |  |  | 0.131 |  |  |  |  |
| Born in Sweden | 1903 (91.9) | 173 (89.2) |  | 1936 (92.1) | 777 (93.7) |  | *Reference* | *Reference* | *Reference* | *Reference* |
| Born outside of Sweden | 167 (8.1) | 21 (10.8) |  | 166 (7.9) | 52 (6.3) |  | 1.38 (0.86-2.24) | 1.21 (0.56-2.75) | 0.78 (0.56-1.08) | 0.98 (0.58-1.64) |
| Rule-breaking |  |  |  |  |  |  |  |  |  |  |
| Truancy^2^ | 1690 (81.7) | 134 (69.4) | <0.001 | 358 (17.0) | 289 (34.9) | <0.001 | 1.97 (1.42-2.28) | 1.23 (0.75-2.01) | 2.61 (2.17-3.13) | 1.96 (1.52-2.55)** |
| Tried hashish/cannabis | 378 (18.3) | 59 (30.6) | 0.005 | 313 (14.9) | 178 (21.6) | <0.001 | 1.56 (1.14-2.15) | 1.27 (0.81-2.00) | 1.57 (1.28-1.92) | 0.90 (0.68-1.20) |
| Weak family |  |  |  |  |  |  |  |  |  |  |
| Low caring parents | 52 (2.5) | 23 (11.9) | <0.001 | 78 (3.7) | 51 (6.2) | 0.004 | 5.23 (3.12-8.75) | 5.61 (2.63-12-05)*** | 1.70 (1.18-2.45) | 0.97 (0.53-1.79) |
| High controlling parents | 255 (12.4) | 62 (32.3) | <0.001 | 167 (8.0) | 149 (18.0) | <0.001 | 3.37 (2.42-4.68) | 2.65 (1.65-4.28)** | 2.54 (2.01-3.23) | 1.03 (1.45-2.84)** |
|  |  |  |  |  |  |  |  |  |  |  |
| Early sexual debut^3^ | 106 (8.2) | 17 (13.5) | 0.046 | 90 (6.6) | 54 (9.1) | 0.046 | 1.74 (1.00-3.00) | 1.24 (0.62-2.49) | 1.43 (1.00-2.03) | 0.72 (0.46-1.12) |
| Sexual abuse^4^ | 148 (7.9) | 29 (16.0) | <0.001 | 455 (22.7) | 350 (44.2) | <0.001 | 2.24 (1.46-3.45) | 1.50 (0.75-3.03) | 2.70 (2.26-3.21) | 2.40 (1.84-3.11)* |
| Penetrating abuse | 43 (2.3) | 9 (5.0) | 0.027 | 119 (5.9) | 128 (16.2) | <0.001 | 2.24 (1.08-4.68) | 1.26 (0.40-3.96) | 3.05 (2.34-3.98) | 1.36 (0.95-1.94) |
| Ever watched pornography |  |  | 0.562 |  |  | <0.001 |  |  |  |  |
| No | 177 (8.6) | 19 (9.8) |  | 1138 (54.4) | 320 (38.6) |  | *Reference* | *Reference* | *Reference* | *Reference* |
| Yes | 1878 (91.4) | 174 (90.2) |  | 955 (45.6) | 508 (61.4) |  | 0.86 (0.52-1.42) | 1.17 (0.42-3.36) | 1.89 (1.60-2.23) | 1.26 (0.96.1,64) |
| If yes: watched deviant pornography^5^ | 234 (12.5) | 30 (17.2) | 0.072 | 61 (6.4) | 66 (13.0) | <0.001 | 1.46 (0.96-2.22) | 1.22 (0.66-2.96) | 2.19 (1.52-3.16) | 1.85 (1.13-3.04) |
| Frequency of watching |  |  | 0.045 |  |  | <0.001 |  |  |  |  |
| Some time each month | 400 (19.6) | 25 (13.0) |  | 257 (12.5) | 143 (17.6) |  | *Reference* | *Reference* | *Reference* | *Reference* |
| Never or once/twice a year | 334 (16.4) | 40 (20.8) |  | 1701 (82.8) | 579 (71.4) |  | 1.92 (1.14-3.22) | 3.42 (1.37-8.51)** | 0.61 (0.49-0.77) | 0.82 (0.62-1.19) |
| Weekly or daily | 1302 (63.9) | 127 (66.1) |  | 97 (4.7) | 89 (11.0) |  | 1.56 (1.00-2.43) | 2.20 (1.11-4.36)** | 1.65 (1.16-2.35) | 1.68 (1.06-2.65)* |

^1 Living with both parents include “alternating between mother and father”, 2 No used as reference^

^3 In 2004 and 2009 the question was “Have you skipped school?”, in 2014 the question was “Have you frequently skipped school?”, 4 Before the age of 14 years^

^5 The question about fondling was more strictly defined in 2009 and 2014 studies. 6 Included violent, animal and child pornography. * Effect size = 0-0.29, ** effect size = 0.3-0.49, effect size ≥0.5^
